# Berberine-Loaded Composite Phospholipid Ethosome Hydrogels: A Therapeutic for Mastitis via Regulating the NF-κB and PI3K/Akt Signaling Pathway

**DOI:** 10.3390/ani16091310

**Published:** 2026-04-24

**Authors:** Binwen Zhang, Zheng Wei, Mei Yang, Xin Wang, Qiang Shan, Zheng Cao

**Affiliations:** Heilongjiang Key Laboratory for Laboratory Animals and Comparative Medicine, College of Veterinary Medicine, Northeast Agricultural University, NO. 600, Changjiang Road, Harbin 150030, China; 13569501808@163.com (B.Z.); m15003567979@163.com (Z.W.); 17513204419@163.com (M.Y.); 17642186580@163.com (X.W.);

**Keywords:** mastitis, berberine, hydrogels, ethosomes, NF-κB, PI3K/Akt

## Abstract

Mastitis is a common disease in dairy cows, mainly caused by Staphylococcus aureus and Escherichia coli. Berberine (BBR) has antibacterial and anti-inflammatory potential, but its application is limited due to poor oral absorption and difficulty in reaching mammary tissue. To address this, this study developed a BBR-loaded composite ethosome hydrogel (BBR-CEH) to achieve targeted mammary delivery through local transdermal administration. The experimental results showed that BBR-CEH has good chemical stability and biosafety. Subsequently, a mouse mastitis model was established by intraductal injection of 50 µL of bacterial mixture (*E. coli:S. aureus* = 1:1, each at 1 × 10^7^ CFU/mL). The results showed that after BBR-CEH treatment, the mRNA expression of *TNF-α* (tumor necrosis factor-alpha), *IL-6* (interleukin-6), and *IL-1β* (interleukin-1 beta) was significantly decreased, the mRNA expression of *ZO-1* (zonula occludens-1), *Occludin*, and *Claudin-4* was significantly increased, and *Bax*/*Bcl-2* (Bcl-2-associated X protein/B-cell lymphoma 2) was significantly reduced (*p* < 0.01), indicating alleviation of mastitis by reducing inflammation, improving tight junctions, and inhibiting apoptosis. Finally, network pharmacology and in vivo experiments confirmed that its mechanism involves the NF-κB (nuclear factor kappa-B) and PI3K/Akt (phosphoinositide 3-kinase/protein kinase B) pathways. Thus, topical BBR-CEH may represent a promising new strategy for mastitis treatment.

## Simple Summary

Mastitis is a common disease in dairy cows, with major pathogens including *Staphylococcus aureus* and *Escherichia coli*. Although berberine has antibacterial and anti-inflammatory properties, it is poorly absorbed after oral administration. We developed a novel berberine-loaded hydrogel that can be applied to the udder, delivering the drug directly to the mammary tissue. Experimental results showed that this hydrogel is stable, safe, and capable of sustained drug release. Tests in a mouse model demonstrated that it effectively reduces bacteria-induced inflammation, protects the mammary barrier, and minimizes cell damage. Its mechanism of action is related to the regulation of intracellular NF-κB and PI3K/Akt signaling pathways. This study suggests that the hydrogel offers a new topical strategy for treating mastitis, solving the problem of poor oral absorption through local application. It holds promise for providing dairy cows with a more convenient and effective treatment option in the future.

## 1. Introduction

Mastitis poses a significant challenge to the dairy cattle industry, hindering the healthy development of dairy farming and the wider dairy industry [1]. Most cases of mastitis are caused by the invasion of pathogenic microorganisms into the mammary tissue, such as *Staphylococcus aureus* (*S. aureus*) and *Escherichia coli* (*E. coli*) [2]. Although antibiotic therapy remains the cornerstone of mastitis prevention and treatment, drug residues and bacterial resistance have become a public problem threatening human health [3]. Berberine (BBR) is an alkaloid found in Chinese herbs, such as *Berberis vulgaris*, *Berberis aquifolium* and *Hydrastis canadensis* [4]. BBR possesses numerous pharmacologic effects, including antibacterial, anti-inflammatory and antioxidant properties [5]. At present, BBR has been proven to effectively reduce bacterial load in breast tissue, alleviate pathological damage, and alleviate inflammatory reactions in rodent models of mastitis such as rats [6]. Compared with conventional antibiotics, berberine (BBR) offers distinct advantages for clinical application, including lower toxicity, reduced risk of drug resistance, and fewer adverse effects [7]. However, its clinical efficacy is limited by poor gastrointestinal absorption, which compromises oral bioavailability. Although intramammary injection of BBR-loaded formulations enables targeted local delivery, this invasive administration route raises concerns regarding teat canal integrity and practical convenience in routine veterinary settings [8]. Therefore, developing alternative delivery strategies that overcome these limitations is essential to fully realize the therapeutic potential of BBR.

Transdermal drug delivery systems (TDDSs) are emerging as a desired delivery system to replace traditional drug delivery routes. The advantages of TDDSs over oral administration and injection include avoiding first-pass effects, reducing injection pain, maintaining stable blood concentrations, and improving patient compliance [9]. However, the barrier function of the stratum corneum (SC) hinders the penetration of most drugs into the skin. Ethosomes are soft, malleable nanovesicles that can alter the regular, tight arrangement of lipid molecules in the SC, thereby helping the drug to penetrate deeper into the skin [10]. Phospholipids are an important component of cell membranes and are commonly used in the preparation of ethosomes. Phosphatidylcholine (PC), an unsaturated phospholipid, is used to prepare most ethosomes. Nevertheless, ethosomes are susceptible to phospholipid peroxidation during storage, and then induce the structural disruption of ethosomes, resulting in a decreased encapsulation efficiency [11]. Hydrogenated phosphatidylcholine (HSPC), a saturated phospholipid, is also used to prepare some ethosomes. Although the emergence of HSPC solved the problem of phospholipid peroxidation, ethosomes prepared using HSPC exhibit a larger particle size, resulting in unsatisfactory skin penetration [12]. Recently, Chen et al. showed that the addition of HSPC not only alleviated the peroxidation reaction of PC, but also improved the stability, drug-carrying capacity and skin penetration of ethosomes [11]. However, the major challenges of ethosomes as liquid formulations include their limited skin retention time and slow-release ability [13]. These challenges can be overcome using hydrogels. Hydrogels are three-dimensional polymer networks that are widely used in topical applications due to their excellent adhesion, swelling behavior, biocompatibility, and slow-release ability [14]. It is noteworthy that ethosomes exhibit high compatibility with hydrogels; incorporating these vesicular carriers into hydrogels enhances their stability and skin permeation capabilities, thereby creating advantageous conditions for TDDSs [15].

**Figure 1:**
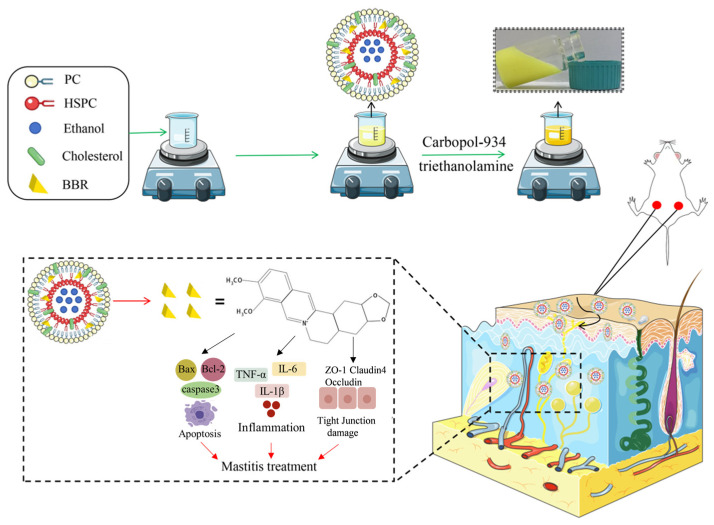
Schematic diagram of BBR-CEH transdermal drug delivery for the treatment of mastitis.

In view of the limitations of a poor oral bioavailability and the invasiveness of intramammary injection, this study aimed to develop an efficient, safe, and stable TDDS for BBR. This strategy enables sustained, localized delivery through the skin, thereby circumventing the drawbacks associated with both oral and intramammary administration. As illustrated in Figure 1, we successfully fabricated berberine-loaded composite phospholipid ethosome hydrogels (BBR-CEH). The physicochemical properties, in vitro antimicrobial activity, and safety of the formulation were systematically characterized. Furthermore, the therapeutic efficacy of BBR-CEH was evaluated in mouse models of mastitis induced by Staphylococcus aureus and Escherichia coli, and the underlying mechanisms were investigated using network pharmacology combined with in vitro cellular assays. Collectively, this study not only provides a novel delivery system for the treatment of mastitis but also offers valuable insights and practical evidence to support the clinical translation of natural product-based therapeutics.

## 2. Materials and Methods

### 2.1. Materials

Phosphatidylcholine (purity ≥ 90%), hydrogenated phosphatidylcholine (purity ≥ 95%) and cholesterol (purity ≥ 99%) were purchased from Shanghai A.V.T. Pharmaceutical Co., Ltd. (Shanghai, China). Berberine (purity ≥ 98%) was purchased from Solarbio Technology Co., Ltd. (Beijing, China). Acetonitrile (purity ≥ 99.9%) of high-performance liquid chromatography (HPLC) grade was purchased from Yuwang Chemical Ltd. (Shandong, China). Tri-ethanolamine (purity ≥ 99%) was purchased from Aladdin Bio-Technology Co., Ltd. (Shanghai, China). Carbopol-934 (average molecular weight of about 3 × 10^6^) was purchased from Macklin Biochemical Technology Co., Ltd. (Shanghai, China). Distilled water was utilized throughout the experiment.

### 2.2. Preparation and Optimization of BBR-Loaded Composite Phospholipid Ethosomes

BBR-loaded composite phospholipid ethosomes (BBR-CE) were prepared using the ethanol injection method. Briefly, BBR was dissolved along with PC, HSPC and cholesterol in an appropriate quantity of anhydrous ethanol. Then, the ethanol solution was slowly added to phosphate-buffered saline (PBS) and spun continuously for 20 min at 700 rpm. To reduce vesicle size, the ethosomes were sonicated using a probe sonicator for 30 s at 4 °C. The BBR-CE was subsequently filtered using a 0.45 μm microporous membrane filter.

Three factors and three levels of an orthogonal test were designed through changing the phospholipid ratio (A), ethanol concentration (B) and cholesterol content (C), and nine different ethosomes were prepared. Analyzing the vesicle size and entrapment efficiency of BBR-CE determined a better preparation process for BBR-CE (Table 1).

### 2.3. Characterization of BBR-CE

#### 2.3.1. Vesicle Size and Zeta Potential

The vesicle size (VS), zeta potential and size distribution of BBR-CE were determined using a sizer Nano ZS (Malvern Instruments, Malvern, UK). The VS distribution was defined as the polydispersity index (PDI).

#### 2.3.2. Morphological Analysis

The morphology of BBR-CE was studied using transmission electron microscopy (TEM). Briefly, a drop of diluted BBR-CE was placed on a copper grid and left to dry. The sample was then fixed using a 1% (*w*/*v*) solution of phosphotungstic acid. Finally, the sample was observed using TEM, and images were captured.

#### 2.3.3. Entrapment Efficiency

The entrapment efficiency (EE) of BBR was determined by an ultrafiltration method. Briefly, 1 mL of BBR-CE was loaded into a Millipore centrifugal filter (100 kDa MWCO) and centrifuged at 4 °C, 3000 rpm for 15 min. To ensure complete recovery of unbound BBR, the retentate was resuspended in 1 mL of fresh methanol, transferred to a new filter unit, and recentrifuged under identical conditions. The combined filtrates were then diluted to 10 mL with methanol, and free BBR was quantified by HPLC. The EE was calculated as:

EE (%) = (W − W_1_)/W × 100%

where W is the total amount of BBR in BBR-CE and W_1_ is the amount of free BBR in the filtrate.

#### 2.3.4. Effect of HSPC on the Peroxidation of PC

The extent of lipid peroxidation was estimated based on malondialdehyde (MDA) concentrations. BBR-CE was prepared by dissolving various phospholipids (PC, HSPC, or mixtures of PC and HSPC) into anhydrous ethanol. The MDA content in BBR-CE was determined at 0, 7, 14 and 28 d using the MDA Assay Kit (Nanjing Jiancheng Bioengineering Institute, Nanjing, China).

### 2.4. Preparation of BBR-CEH

The composition of different hydrogel formulation is listed in Table 2. In brief, accurately weighed amounts of carbopol-934 (0.5% *w*/*w*, 1% *w*/*w* and 1.5% *w*/*w*) were dissolved in BBR-CE or free drug until a smooth, lump-free and homogeneous hydrogel was obtained. The pH was adjusted by adding an appropriate amount of triethanolamine during BBR-CEH preparation.

### 2.5. Characterization of BBR-CEH

#### 2.5.1. Homogeneity Observations and pH Measurement of BBR-CEH

The prepared BBR-CEH was visually checked for homogeneity and smoothness. The pH was measured by using a digital PB-100 pH meter (Sartorius, Shanghai, China) to ensure that the formulation was not a skin irritant.

#### 2.5.2. Viscosity

The prepared BBR-CEH was analyzed for viscosity. The viscosity of the different BBR-CEH groups was measured at room temperature using an NDJ-1 digital viscometer (LiChen, Shanghai, China) with a No. 4 rotor.

#### 2.5.3. Spreadability

The prepared BBR-CEH was analyzed for spreadability. A total of 500 mg of the prepared BBR-CEH was placed between two glass slides and weighted down for 1 min. Finally, the diameter of the diffusion was measured using a scale.

#### 2.5.4. Drug Content

A total of 500 mg of BBR-CEH was weighed out precisely and fixed in 10 mL of methanol. The mixture was stirred for 1 h until the drug was completely dissolved. The drug content was determined by the HPLC method.

#### 2.5.5. Fourier Infrared Spectroscopy (FTIR) Study

A Nicolet IS5 Fourier Infrared Spectrometer (Thermo Fisher Scientific Inc., Waltham, MA, USA) was used for infrared detection of samples, maintaining a scanning range of 400–4000 cm^−1^ with a resolution better than 0.09 cm^−1^.

#### 2.5.6. In Vitro Drug Release Study

HPLC was used to analyze BBR, with acetonitrile–phosphoric acid as the mobile phase for gradient elution and a reversed-phase C18 column, and method validation was performed. Details are provided in TEXT S1. In vitro drug release studies of BBR-CE (H_4_) and BBR-CEH (H_2_) were performed using the dialysis bag method. Before the study, the dialysis membrane was pretreated to fully wet the membrane. The samples were completely transferred into the sealed dialysis membrane. An amount of 200 mL of saline was selected as the release medium at 37 ± 0.5 °C and stirred with a magnetic bar at 200 rpm. Samples (1 mL) were gathered at various time points and then an equal volume of fresh medium was added. The concentration of BBR in the release medium at different time points was determined by HPLC.

#### 2.5.7. Ex Vivo Skin Permeation Study

Ex vivo permeation studies of BBR-CEH (H_2_) and the conventional hydrogels (H_5_) were performed using a modified Franz diffusion cell. After the female mice (10 weeks old, weighing 36–39 g) were sacrificed, the intact skin of the mouse abdomen was cut off for study. The skins were subsequently washed three times with saline and stored below −18 °C to maintain their metabolic efficiency.

The skins were clamped between the donor and recipient compartments of the modified Franz diffusion cell. The receptor solution, consisting of 40 mL of saline, was kept at 37 ± 0.5 °C and stirred with a magnetic bar at 200 rpm. H_2_ and H_5_ were placed on the skin surface of the donor compartment. At predetermined time intervals (1, 2, 4, 6, 8, 12, 24 h), 5 mL of receptor solution sample was removed and refilled with the same volume of receptor solution again. The concentration of BBR in the receptor solution was analyzed by HPLC. The percentage of drug permeated was calculated using the equation.

$$\mathrm{Percentage}\;\mathrm{drug}\;\mathrm{permeated}=\frac{\mathrm{Amount}\;\mathrm{of}\;\mathrm{drug}\;\mathrm{permeated}\;\mathrm{at}\;a\;\mathrm{time}}{\mathrm{Total}\;\mathrm{amount}\;\mathrm{of}\;\mathrm{drug}}\times \;100\%$$

#### 2.5.8. Hemolysis Assay

Excellent biocompatibility is essential for biomaterials. A hemolysis assay was used to evaluate the hemocompatibility of BBR-CEH. Red blood cells were extracted from blood by 15 min centrifugation at 2000 rpm at low temperature. Subsequently, red blood cells were diluted using PBS to achieve a 4% volume concentration. Triton X-100 (Solarbio, Beijing, China) was used as the positive standard and PBS as the negative standard. The red blood cell suspension was mixed with BBR-CEH extracts (750 µg/mL and 1000 µg/mL) and incubated for 3 h at 37 °C. The mixture was then centrifuged at 3000 rpm for 10 min. Finally, 200 μL of the supernatant was seeded in a 96-well plate, and the absorbance was measured at 540 nm by a microplate reader.

$$\mathrm{Hemolysis}\;\mathrm{assay}\;(\%)=\frac{{\mathrm{OD}}_{S}-{\mathrm{OD}}_{N}}{{\mathrm{OD}}_{P}-{\mathrm{OD}}_{N}}\times \;100\%$$

where OD_N_, OD_S_, and OD_P_ are the absorbance values of the negative control, test sample, and positive control, respectively.

#### 2.5.9. Antibacterial Activity Study

In our previous study, the minimum bactericidal concentration (MBC) of BBR for *S. aureus* and *E. coli* was 500 μg/mL. In the present study, the antimicrobial activity of BBR-CEH was evaluated by the microdilution method. The final concentration of BBR-CEH was set at to 250 µg/mL, 500 µg/mL, 750 µg/mL and 1000 µg/mL. An amount of 1 mL of different concentrations of BBR-CEH was added to a 5 mL sterile test tube using a sterile syringe. Then either 1 mL of *S. aureus*, *E. coli*, or mixed bacterial (1:1) was added. Blank composite phospholipid ethosome hydrogels (B-CE control) and Luria–Bertani (LB) medium containing bacteria (LB control) were used as controls. The mixture was incubated in a 37 °C incubator for 24 h. Then, 100 µL of the mixture was spread on solid agar medium and incubated for 24 h to observe the number of colonies. The lowest drug concentration resulting in fewer than five colonies was the MBC.

#### 2.5.10. Skin Irritation Study

A total of 18 female mice (10 weeks old, weighing 36–39 g) were randomly divided into an intact skin group and a broken skin group by using the left–right comparison method for skin on both sides of the same body. Then, mice were depilated on their backs. Low (500 μg/mL), medium (750 μg/mL) and high (1000 μg/mL) doses of BBR-CEH were applied to the left side of the skin. Saline was applied on the right side as a control group. Twice daily for 14 d, erythema and edema were observed 1 h, 24 h, 48 h, 72 h after removal of the drug. The levels of redness, swelling, and irritation were rated on a scale of 0 to 4, and the scoring criteria are shown in TEXT S2.

### 2.6. Animal Experiments and Histological Analysis

#### 2.6.1. Animal Experiments

In this experiment, 30 female Kunming mice, 10 weeks old and weighing 37–41 g at 3 days after delivery, were purchased from the Experimental Animal Center of the Second Affiliated Hospital of Harbin Medical University. Mice were randomly divided into three groups (*n* = 10 per group): control group (CG), model group (MG), and treatment group (CTG). Randomization was performed using a random number table method. All animal experiments were authorized by the Institutional Animal Care and Use Committee of Northeast Agricultural University (NEAUEC22303129). During the experiment, the animal grouping was blinded to the operator and outcome evaluator. Infection models of mastitis were constructed in the 30 female Kunming mice via the reported method. The fourth pair of mammary glands and surrounding skin of mice were disinfected with 75% ethanol. The tip of the nipple was then removed 1 mm from the peak to expose the teat ducts. The teat duct of CG mice was injected with 50 μL of sterile saline. Other mice were injected with 50 μL of the bacterial mixture (*E. coli*:*S. aureus* = 1:1, each at 1 × 10^7^ CFU/mL) via the teat duct [16]. After 24 h, mice were observed for signs of clinical relevance, such as mental status, appetite and mammary gland swelling. After the successful construction of the mastitis model, BBR-CEH was applied to mammary skin in CTG mice once daily for 5 d.

Five days later, the mice were euthanized by cervical dislocation. A portion of mammary tissue was collected and fixed in 4% paraformaldehyde for subsequent histopathological examination, while the remaining mammary tissue was stored at −80 °C.

#### 2.6.2. FITC-Albumin Permeability in the Mammary Gland

The integrity of the blood–milk barrier (BMB) in the mammary gland was observed. After euthanizing the mice, the fourth mammary gland was collected and immersed in 2 mL of PBS containing 3 mg/mL of FITC-albumin for 10 min, ensuring that the interstitial side is fully submerged. After FITC-albumin treatment, the mammary glands were washed three times in PBS and then immersed in PBS containing 4% paraformaldehyde for 15 min. The pre-processed breast tissue was embedded in the optimal cutting temperature (OCT) compound and frozen with liquid nitrogen, and 5 μm frozen sections were obtained. The frozen sections were stained with 4′,6-diamino-2-phenylindole (DAPI) and the images were observed under confocal laser scanning microscopy. Images were captured at the same parameters and correct background fluorescence.

#### 2.6.3. Histopathological Examination

The mammary glands of female mice were collected and fixed in a 4% formaldehyde solution for paraffin embedding, sectioning, dehydration, transparency, and sealing. Hematoxylin eosin (HE) staining was performed. The prepared slices were placed under a microscope and scanned using a digital section scanner to observe histopathological changes. Meanwhile, histopathological changes were scored [17], and the detailed scoring criteria are provided in the TEXT S3.

#### 2.6.4. Ultrastructure Observation

Firstly, the freshly isolated breast tissue were immersed in freshly prepared, pre-cooled glutaraldehyde fixative. Then, the organization underwent dehydration and other processing steps. Finally, photos were taken using a transmission electron microscope (H-7650, Hitachi, Tokyo, Japan).

#### 2.6.5. Immunofluorescence (IF)

Mouse mammary gland sections were incubated with a ZO-1 primary antibody (1:1000, Servicebio, Wuhan, China). After washing with PBS, the sections were incubated with an appropriate fluorescently labeled secondary antibody. The nuclei were then counterstained with DAPI (Servicebio, Wuhan, China). Finally, the samples were imaged using fluorescence microscopy.

#### 2.6.6. Terminal Deoxynucleotidyl Transferase dUTP Nick Labeling (TUNEL) Assay

Apoptotic cells in mouse mammary gland sections were detected using a TUNEL Assay Kit (Beyotime, Shanghai, China, C1088) according to the manufacturer’s instructions. Following the TUNEL reaction, the sections were rinsed with PBS and nuclei were counterstained with DAPI (Servicebio, Wuhan, China). Images were captured using fluorescence microscopy.

#### 2.6.7. Caspase-3 Activity Assay

Mammary tissue samples were homogenized in ice-cold lysis buffer and centrifuged at 10,000× *g* for 10 min at 4 °C. The supernatant was collected, and protein concentration was determined using a BCA protein assay kit (Beyotime, Shanghai, China), following the manufacturer’s instructions. For Caspase-3 activity measurement, 50 μg of protein from each sample was incubated with the specific substrate using a commercial colorimetric kit (Nanjing Jiancheng, Nanjing, China, Cat.) at 37 °C for 2 h. The absorbance was measured using a microplate reader at 405 nm.

### 2.7. Network Pharmacology

This study obtained relevant target information through a joint retrieval system that searched multiple databases. Mastitis-related targets were identified through the OMIM, GeneCards and CTD databases, while BBR-related targets were acquired through the SuperPred and SwissTargetPrediction databases. Following this, the common targets were imported into the STRING database to generate a protein–protein interaction (PPI) network, and the resulting network was visualized using Cytoscape 3.8.0. Finally, GO and KEGG enrichment analyses were conducted for the BBR-related targets using the DAVID database.

### 2.8. Molecular Docking

The PubChem database was used to download the compound’s SDF file and the Protein Data Bank (PDB) database was used to download the core target protein structure file in PDBQT format. AutoDock Vina software 1.5.7was then used to adjust the size and position of the receptor’s active pocket for molecular docking studies. The results were converted into PDB format using the OpenBabel 3.1.1 software package and visualized using PyMOL 3.0.3.

### 2.9. Western Blotting

The relative levels of target proteins were determined by Western blotting. For additional details, refer to TEXT S4.

### 2.10. Quantitative Real-Time Polymerase Chain Reaction (QRT-PCR)

QRT-PCR was used for the relative quantification of mRNA. Primers used are shown in Table S3. For additional details, refer to TEXT S5.

### 2.11. Statistical Analysis

All experiments were independently repeated three times (*n* = 3). The values are presented as the mean ± standard deviation (SD). For comparisons among multiple groups, one-way analysis of variance (ANOVA) was performed. If the data met the assumptions of normality and homogeneity of variance, post hoc pair-wise comparisons were conducted using the least significant difference (LSD) test. If homogeneity of variance was violated, Dunnett’s T3 test was applied. For comparisons between two groups, Student’s *t*-test was used. Statistical analyses were performed using SPSS software (version 22.0, IBM Corp., Armonk, NY, USA). We considered *p* < 0.05 as significant and *p* < 0.01 as markedly significant.

## 3. Results

### 3.1. Subsection

#### 3.1.1. Optimization of BBR-CE Formulations

The results of the orthogonal experimental results are presented in TEXTs S6 and S7; the VS and EE values of BBR-CE were between 169.4 and 447.4 nm and from 91.48 to 97.97%, respectively. Intuitive analysis demonstrated that the influence of each factor on VS was in the order of A > C > B based on the magnitude of the R-value. The results of the three-factor analysis showed that the optimal parameters for BBR-CE were A1B2C2 (TEXT S6). Additionally, the influence of each factor on EE was found to be in the order A > C > B. The results of the three-factor analysis showed that the optimal parameters for BBR-CE were A3B2C2 (TEXT S7) because the ideal size of ethosomes suitable for transdermal drug delivery is less than 300 nm. The VS of BBR-CE at phospholipid ratio A3 was 426.4–447.4 nm, indicating that it is not suitable for transdermal drug delivery. Meanwhile, factor A has no significant effect on EE. Consequently, the final optimal formulation was selected as phospholipid ratio (1:1, A1), ethanol concentration (30%, B2) and cholesterol content (30 mg, C2).

#### 3.1.2. Characterization of BBR-CE

**Figure 2:**
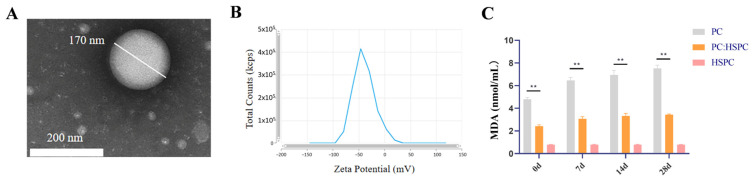
(**A**) TEM image of optimized BBRCE. (**B**) The zeta potential distribution map of BBRCE. (**C**) The effect of HSPC on PC MDA content was determined (*n* = 3). ** *p* < 0.01 versus the PC:HSPC value.

Using the optimal formulation, three batches of BBR-CE were prepared for characterization. The measured VS was 169.4 ± 3.1 nm (Figure 2A). The particles appear as uniformly dispersed spheres. The PDI was 0.24 ± 0.04, and the zeta potential was −30.91 ± 1.31 mV (Figure 2B). The EE was 95.76 ± 2.39%.

To evaluate the effect of HSPC on phospholipid peroxidation, the MDA content was determined. At 0 d, 7 d, 14 d, and 28 d, the MDA content was highest in the presence of pure PC, reaching about 7.52 nmol/mL, indicating the most severe lipid peroxidation occurred under these conditions. In contrast, after mixing PL with HSPC, the MDA content significantly decreased, with each group approaching 3.43 nmol/mL (*p* < 0.01), indicating effective inhibition of lipid peroxidation. Pure HSPC had the lowest MDA content, about 0.78 nmol/mL, which further confirms the good antioxidant effect of the selected formula (Figure 2C).

#### 3.1.3. Characterization and Performance of BBR-CEH

##### Characterization of BBR-CEH

Table 3 summarizes the physicochemical properties of BBR-CEH formulated with different prescriptions. All formulations exhibited excellent homogeneity and smoothness. The pH values ranged from 5.81 to 6.02. The viscosities of H_1_ and H_2_ are 39.80 ± 2.02 Pa·s and 79.20 ± 1.50 Pa·s, respectively, both demonstrating good spreadability. However, the viscosity of H_3_ exceeds 100 Pa·s, and its excessively high viscosity results in poor spreadability, indicating that it may not be suitable for transdermal drug delivery. In contrast, although H_1_ exhibits good spreadability, its relatively low viscosity may be unfavorable for drug adhesion. Taking all these factors into consideration, we selected the H_2_ formulation with moderate viscosity for further study.

##### Performance of BBR-CEH

- FTIR study

**Figure 3:**
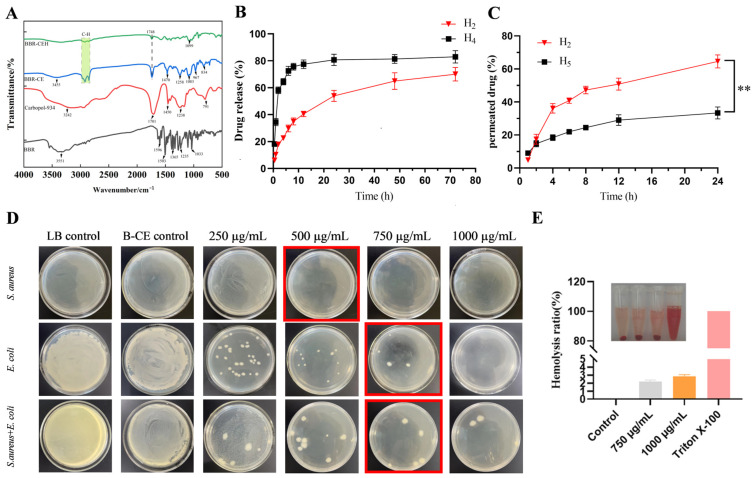
(**A**) Fourier transform infrared spectra of different samples. (**B**) In vitro drug release studies of BBRCEH (H_2_) formulation and BBRCE (H_4_) suspension (*n* = 3). (**C**) In vitro skin permeation profiles of BBRCEH (H_2_) and conventional hydrogel (H_5_) (*n* = 3). (**D**) In vitro bacteriostatic effect of BBR-CEH. Note: Red box indicates the MBC of the BBRCEH. (**E**). Hemolysis rate of BBRCEH. ** *p* < 0.01 versus the H_5_ value.

FTIR was analyzed for BBR, BBR-CE, carbopol-934 and BBR-CEH (Figure 3A). BBR showed a characteristic peak at 1235 cm^−1^–1365 cm^−1^. However, these characteristic peaks appeared to shift or disappear in BBR-CE, probably due to the successful encapsulation of BBR. In addition, the O-H stretch of BBR-CE at around 3455 cm^−1^ is widened, indicating that hydrogen bonds are formed [18]. In BBR-CEH, there was variation in peak intensity and displacement of carbopol-934 near 1450 cm^−1^ (-C-C vibration) [19], possibly owing to the formation of a hydrogel network. Moreover, BBR-CE showed characteristic peaks of C–H stretching (2850 cm^−1^ to 2919 cm^−1^). In the prepared BBR-CEH, these peaks disappeared or shifted, possibly due to the successful encapsulation of the ethosomes in the hydrogels. In summary, the results indicated that the components of BBR-CEH had good compatibility and that BBR was successfully encapsulated into ethosomes and further stably loaded into the hydrogel network.

- In vitro drug release study

The release behavior of BBR in BBR-CE (H_4_) and BBR-CEH (H_2_) was studied over 72 h (Figure 3B). The results showed that H_4_ released the majority of the drug within the first 6 h, with a cumulative release rate of approximately 75% after 6 h, after which the release rate leveled off, ultimately reaching about 80%. In contrast, H_2_ released about 20% to 25% of the drug in the initial 4 h, after which it exhibited a sustained slow-release pattern, reaching a cumulative release rate of 70.00 ± 5.01% at 72 h. These findings indicate that BBR-CEH possesses a better slow-release ability compared to BBR-CE.

- Ex vitro skin permeation study

An ex vitro skin permeation study was conducted on BBR-CEH (H_2_) and the conventional hydrogel (H_5_) over a period of 24 h (Figure 3C). The results showed that there was no significant difference between H_2_ and H_5_ after 2 h, but there was a significant difference after 4 h (*p* < 0.01). The cumulative permeation percentages of H_2_ and H_5_ after 24 h were 64.60 ± 3.98% and 33.37 ± 1.14%, respectively, indicating that the drug penetration through mouse skin from H_2_ was significantly higher than that from H_5_ (*p* < 0.01).

- Antibacterial activity study

Using the microdilution method, this study assessed the concentration-dependent bactericidal activity and minimum bactericidal concentration (MBC) of BBR-CEH against *S. aureus*, *E. coli*, and their 1:1 mixed culture. It was determined that the MBC of BBR-CEH was 750 μg/mL against *Staphylococcus aureus*, *Escherichia coli*, and the 1:1 mixed bacterial culture (Figure 3D). In contrast, the blank vehicle control showed no antibacterial effect. Therefore, BBR-CEH at a drug concentration of 750 µg/mL was selected for subsequent experiments.

- Hemocompatibility study

As shown in Figure 3E, compared with the positive control, the color change in all tested supernatants was minimal, indicating negligible hemolysis. Furthermore, all the hemolysis rates were below 3%, which is lower than the acceptable threshold of 5%. These results demonstrate the hydrogel’s good blood compatibility.

- Skin Irritation Study

The skin irritation study demonstrated that BBR-CEH at various concentrations did not induce edema, erythema, or any other irritation reactions on either intact or damaged skin at 1, 24, 48, and 72 h after administration. The irritation scores at all time points were zero (TEXT S8). These results indicate that BBR-CEH exhibits no skin irritancy at low, medium, and high doses, demonstrating good biosafety. Therefore, a concentration of 750 µg/mL was selected for subsequent mastitis treatment experiments.

#### 3.1.4. Effect of BBR-CEH on Inflammation, TJ Damage and Apoptosis in Mouse Model of Mastitis

**Figure 4:**
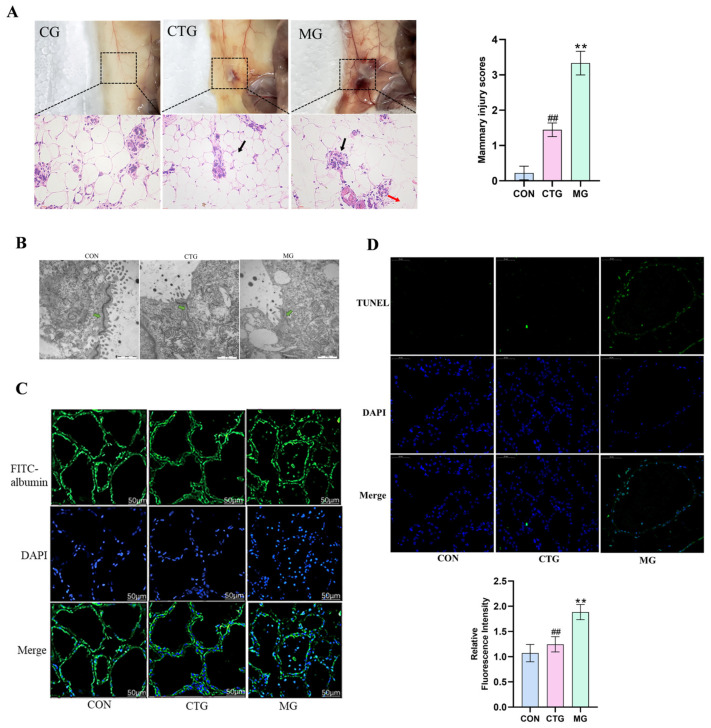
(**A**) Morphological observation and H&E staining of mouse mammary tissue. Note: black arrows represent mammary follicles; red arrows represent inflammatory infiltrates. The scoring criteria vary from 0 points to 4 points, corresponding to no injury, mild injury, moderate injury, severe injury, and extreme injury, respectively (*n* = 3). (**B**) TEM image, TJ (green arrows) (*n* = 3). (**C**) BMB permeability of mammary tissue and FITC albumin localization. (**D**) Illustrative depictions of TUNEL fluorescence. Cell nucleus (blue); DNA fragments (green). Scale bar: 50 μm (*n* = 3 ** *p* < 0.01 versus the CON value, ## *p* < 0.01 vs. the MG value.

The results of the histopathological analysis showed no significant pathological changes or inflammatory infiltration in the mammary tissue of the CG mice (Figure 4A). In contrast, the mammary tissue of the MG showed severe pathological damage, including thickening of the alveolar wall and increased inflammatory cell infiltration. Meanwhile, we found that the histopathological damage to the mammary tissue of the CTG was significantly alleviated: inflammatory infiltration was reduced and the tissue structure was more intact. Histopathological scoring revealed minimal injury in the CG, extreme damage in the MG, and significantly alleviated damage in the CTG group (intermediate between CG and MG), with both comparisons showing significant differences (*p* < 0.01). Electron microscopy results showed (Figure 4B) that BBR-CEH significantly alleviated TJ injury. Meanwhile, we evaluated the leakage of FITC-albumin from the interstitial side into the alveolar lumen. The results showed that FITC-albumin was clearly localized on the interstitial side in the mammary tissue of CG mice (Figure 4C). However, compared to the CG, FITC-albumin was also present in the mammary alveolar lumen, indicating BMB disruption. As expected, leakage of FITC-albumin into the mammary alveolar lumen was reduced in the mammary tissue of CTG mice, indicating that BMB disruption was mitigated. TUNEL staining showed an increase in nuclear DNA fragmentation in the MG, indicating a significant induction of cell apoptosis. The addition of BBR-CEH significantly reduced this damage (Figure 4D).

#### 3.1.5. Network Pharmacology and Molecular Docking

**Figure 5:**
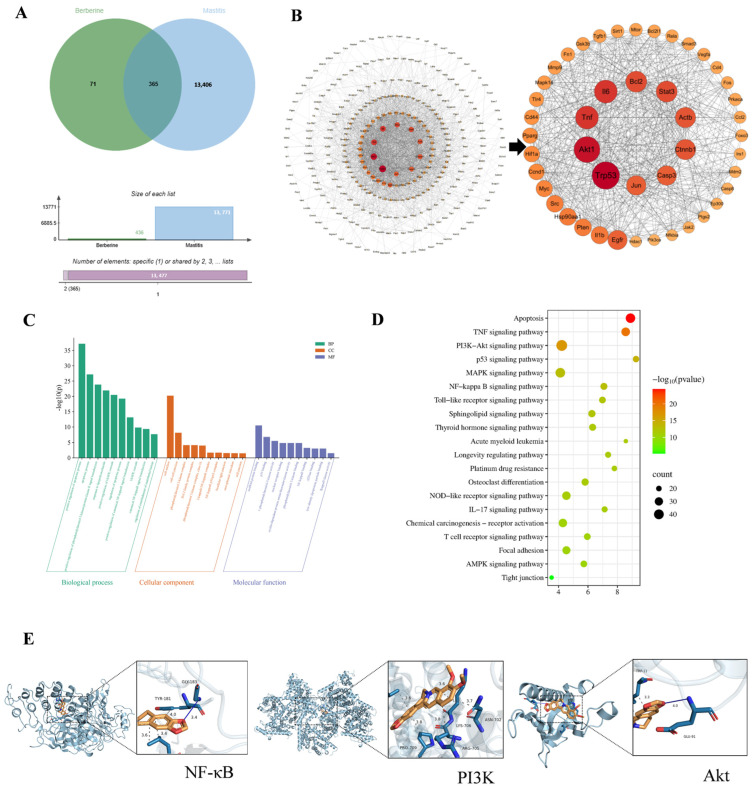
(**A**) Venn diagram illustrating shared targets between BBR and mastitis. (**B**) Proteinprotein interaction network of core targets. Node size and color intensity represent degree centrality. (**C**) GO analysis of BBR for mammary inflammation. (**D**) KEGG pathway analysis of BBR against mastitis. (**E**) Schematic diagram of the docking of BBR with NFκB, PI3K, Akt molecules.

Through database screening, a total of 436 BBR-related targets and 13,771 mastitis-related targets were obtained, and the intersection yielded 365 potential therapeutic targets (Figure 5A). PPI network analysis revealed a final network consisting of 300 nodes and 2265 edges (Figure 5B). These genes were identified as potential key targets involved in mastitis and selected for subsequent functional enrichment and network topology analysis (Figure 5C,D). GO analysis shows that the protective effect of BBR on mastitis is related to NF-κB, PI3K signaling pathways, cell apoptosis, and TJ. KEGG pathway analysis shows that breast injury is closely related to inflammation and cell apoptosis, involving key signaling pathways such as the PI3K/Akt signaling pathway, apoptosis pathway, p53 signaling pathway, NF-κB signaling pathway and tight junction (TJ) signaling pathway. Molecular docking analysis was conducted to investigate the binding ability and interaction mode of BBR with p65, PI3K, and Akt. The results showed that BBR exhibited good binding affinity with all three target proteins, with binding energies of −7.7 kcal/mol (p65), −8.6 kcal/mol (PI3K), and −6.3 kcal/mol (Akt), all below the set effective binding threshold (≤−5.0 kcal/mol). In terms of interaction mode, p65 formed a hydrogen bond with the GLY-183 residue of BBR, with a bond length of 3.4 Å; hydrophobic interactions existed between PI3K and BBR at residues such as ASN-702 and LYS-706; and Akt formed a hydrogen bond with the GLU-91 residue of BBR, with a bond length of 4.0 Å. These results suggest that BBR may exert regulatory effects by binding to these targets (Figure 5E).

#### 3.1.6. Effect of BBR-CEH on NF-κB and PI3K/Akt Signaling Pathways in Mouse Model of Mastitis

**Figure 6:**
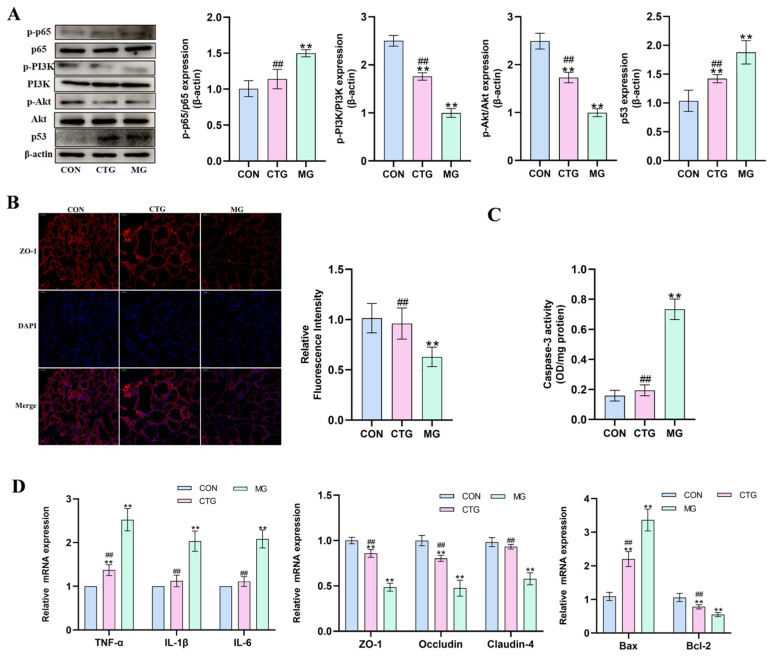
(**A**) The effect of BBR-CEH on NF-κB pathway- and PI3K/Akt pathway-related proteins in mouse mammary gland tissue. (**B**) Representative image of ZO-1 periplasmic localization in mouse mammary gland tissue. ZO-1 (red), nucleus (blue). Scale bar: 50 μm (*n* = 3). (**C**) Caspase-3 activity (*n* = 3). (**D**) The effect of BBR-CEH on the mRNA expression levels in mouse mammary gland tissue (*n* = 3). ** *p* < 0.01 versus the CON value, ## *p* < 0.01 vs. the MG value.

As shown in Figure 6A, compared with the CG, the p-p65/p65 ratio in the MG was significantly increased, while the p-PI3K/PI3K and p-Akt/Akt ratios were significantly lower. Furthermore, p53 expression was significantly increased. Following BBR-CEH treatment, these abnormal indicators were markedly suppressed (*p* < 0.05 or *p* < 0.01). IF showed a significant decrease in ZO-1 signal and blurred membrane localization in the MG, which was significantly restored after treatment with BBR-CEH (Figure 6B). As shown in Figure 6C, compared with the CON group, the Caspase-3 activity in the MG was significantly increased, which was effectively reversed by BBR-CEH intervention. Figure 6D shows that, compared with the CON group, the mRNA expression levels of the pro-inflammatory factors *TNF-α*, *IL-6* and *IL-1β* in the mammary gland tissue of the MG were significantly increased. Additionally, the mRNA expression of TJ protein-related genes *ZO-1*, *Occludin* and *Claudin-4* in the MG was significantly downregulated. Meanwhile, the results of apoptosis-related factor detection showed that in the MG group, the mRNA level of the pro-apoptotic gene *Bax* was significantly increased, while the mRNA level of the anti-apoptotic gene *Bcl-2* was significantly decreased. BBR-CEH intervention significantly reversed the above phenomena.

## 4. Discussion

Mastitis is primarily caused by pathogenic microorganisms such as *S. aureus* and *E. coli*, which makes it one of the most serious diseases endangering the dairy farming industry [20]. Although antibiotic treatment is currently effective, it can easily lead to issues of drug residues and antimicrobial resistance [21]. Plant-derived drugs have attracted attention due to their low cost, low toxicity, and low tendency to induce resistance. However, their complex composition limits their clinical application [22]. BBR, as a single active ingredient derived from plants, has been proven to possess antibacterial and anti-inflammatory effects, showing promise for treating mastitis [23]. However, challenges remain regarding its clinical application due to constraints such as the route of administration, formulation technology, and bioavailability [24]. Therefore, there is an urgent need to develop new formulations that can serve as alternatives to antibiotics. TDDS is emerging as an efficient, safe, and convenient route for treating bovine mastitis, as it avoids the gastrointestinal irritation and first-pass effect associated with oral administration, while also reducing the mechanical damage and labor intensity caused by mammary injection [25].

This experiment involved screening the optimal BBR-CE formulation through an orthogonal design. The physicochemical performance and quality control level of the preparation were evaluated by measuring the particle size distribution, stability and drug loading efficiency [26]. The results showed that the optimized BBR-CE had a VS of 169.4 ± 3.1 nm, which falls within the ideal range (50–300 nm) for nanocarriers used in transdermal drug delivery [27], facilitating close contact with the stratum corneum and promoting drug penetration. Its PDI was 0.24 ± 0.04 (<0.3), indicating a uniform particle size distribution in the system [28]. This ensures physical homogeneity during storage and use, and enhances diffusivity and skin adhesion in practical applications, thereby improving the reliability of transdermal delivery. The zeta potential reached −30.91 ± 1.31 mV, exceeding the commonly used stability threshold (−30 mV) [29], which demonstrates sufficient electrostatic repulsion between particles, effectively preventing aggregation and ensuring the physical stability of the preparation during storage and use. Additionally, the formulation exhibited excellent drug-loading performance, with an EE of 95.76 ± 2.39%, reflecting efficient drug encapsulation capacity, which contributes to enhanced delivery efficiency and reduced drug loss. Thus, the optimized BBR-CE demonstrated favorable characteristics in terms of key quality attributes and met the design requirements for an efficient transdermal drug delivery system.

Based on the optimized BBR-CE, BBR-CEH was further developed with suitable viscosity to ensure satisfactory spreadability and skin retention [30,31]. FTIR analysis indicated good compatibility between BBR and the gel matrix, as evidenced by the shift in or disappearance of characteristic BBR peaks [32]. Such interactions may contribute to the physical stability of the formulation and support efficient drug loading and sustained release. BBR-CEH exhibited sustained release over 72 h, with a cumulative release of 70.00 ± 5.01%, and showed a more stable release profile than conventional gels [33]. This behavior may be attributed to the combined controlled-release effects of the ethosomal nanocarrier and gel network, which could help maintain effective local drug concentrations, reduce dosing frequency, and improve treatment compliance. Simultaneously, skin permeation results showed that the cumulative permeation rate of BBR-CEH (64.60 ± 3.98%) was significantly higher than that of the traditional hydrogel (33.37 ± 1.14%, *p* < 0.01) [34]. The mean particle size of BBR-CEH (169.4 nm) is considered favorable for transdermal delivery through intercellular pathways of the stratum corneum and hair follicles. However, since the permeation study was performed using mouse abdominal skin, caution is needed when extrapolating these findings to bovine mastitis. Bovine skin is generally 2–3 times thicker and has a stronger barrier function due to its denser stratum corneum and different hair follicle distribution [35]. Nevertheless, ethosomes may still retain transdermal potential in bovine skin, as ethanol can disrupt stratum corneum lipid organization while deformable phospholipid vesicles facilitate penetration across the skin barrier [36]. This advantage is not only related to the nanoscale size and surface properties of BBR-CE itself but also benefits from the moisturizing and penetration-enhancing effects of the gel matrix on the stratum corneum [37]. This finding aligns with the potential of “nanocarrier–gel composite systems” in promoting transdermal drug delivery efficiency emphasized in recent studies, further validating the design rationale of BBR-CEH in enhancing transdermal drug transport. Furthermore, antibacterial experimental results demonstrated that BBR-CEH significantly inhibited the growth of *S. aureus*, *E. coli*, and their mixed cultures. Compared to single drug solutions or conventional gels, BBR-CEH exhibited stronger and more prolonged antibacterial effects. This may be attributed to its sustained-release properties, which maintain a higher drug concentration locally at the infection site, thereby controlling bacterial proliferation at the source and reducing infection-related inflammatory responses. This highlights its potential application in the treatment of infectious skin diseases [38]. Safety assessment further confirmed the good biocompatibility of this formulation: BBR-CEH at all tested concentrations caused no skin irritation during the experimental period, and its hemolysis rate was below 3%, meeting the safety standards for medical materials [39]. These results suggest that BBR-CEH not only performs excellently in vitro, but is also safe to use on the skin, providing support for subsequent in vivo studies and clinical use.

Given these findings, BBR-CEH not only achieves synergistic enhancement of release and permeation through structural design and dosage form integration but also possesses distinct antibacterial activity and excellent biocompatibility. These characteristics enable it to provide a novel formulation solution for local skin therapy, particularly for diseases associated with infection risks. Compared with conventional oral berberine formulations, BBR-CEH bypasses gastrointestinal degradation and hepatic–intestinal first-pass metabolism, which are major contributors to the extremely low oral bioavailability of berberine [40]. In addition, the ethosomal hydrogel system combines the penetration-enhancing effect of ethanol with the deformability of phospholipid vesicles, thereby promoting transdermal transport and local retention of the drug in target tissue [10,41]. Relative to injectable administration, BBR-CEH provides a noninvasive route of delivery that is more suitable for repeated local treatment [42]. Moreover, transdermal delivery supports sustained drug release and reduces unnecessary systemic exposure, which is particularly advantageous for localized inflammatory lesions such as mastitis [43].

Mastitis is an inflammatory disease of the mammary tissue caused by pathogenic microorganisms such as *S. aureus* and *E. coli* [44]. After invading the mammary ducts or alveoli, these pathogens can activate local mammary epithelial cells and related immune cells, triggering a series of immune responses that ultimately lead to damage to the mammary tissue [45]. The present study found that *S. aureus*- and *E. coli*-induced mastitis leads to mammary tissue damage and inflammatory cell infiltration, which is consistent with existing research findings [46]. Meanwhile, the FITC-albumin permeability assay and TEM analysis revealed that *S. aureus*- and *E. coli*-induced mastitis resulted in the leakage of FITC-albumin into the interstitial space and disruption of intercellular junction structures, indicating increased paracellular permeability and dysfunction of the BMB. However, after treatment with BBR-CEH, the inflammatory response in mammary tissue was significantly suppressed, and structural tissue damage was also ameliorated. TUNEL staining and the TEM results further indicated that BBR-CEH could reduce apoptosis of mammary epithelial cells and prevent structural damage to TJs. These results are consistent with the multi-target protective effects of BBR that are already known. A previous study has indicated that BBR can alleviate inflammation by inhibiting signaling pathways such as NF-κB, enhance epithelial barrier function, and suppress cell apoptosis, which aligns with the findings of this study [47]. Furthermore, this study demonstrates that BBR-CEH can effectively promote the transdermal penetration of BBR, thereby providing protection against inflammation-related damage in mastitis.

Based on the above findings, BBR alleviates bacterial-induced damage to mammary tissue in cases of mastitis by inhibiting inflammation, reducing apoptosis, and maintaining TJs. However, the exact mechanism by which it acts has not yet been fully elucidated. Therefore, this study employed a network pharmacology approach for systematic analysis. GO analysis revealed that the protective effect of BBR on the mammary gland is closely related to key biological processes such as NF-κB signal transduction, apoptosis, and TJs. KEGG pathway enrichment analysis further demonstrated that BBR mitigates bacterial invasion-induced mammary tissue damage primarily by regulating multiple key signaling pathways, including the PI3K/Akt pathway, apoptosis pathway, p53 pathway, NF-κB pathway, and TJ-related pathways. A previous study has indicated that the NF-κB pathway is not only an inflammatory regulatory pathway but also a critical pathway linking BMB function [48]. Meanwhile, the PI3K/Akt pathway serves as a central hub for regulating processes such as p53 signaling and apoptosis [49]. Notably, NF-κB and the PI3K/Akt signaling pathway do not function independently; crosstalk exists between them. PI3K/Akt can promote nuclear translocation and transcriptional activity of NF-κB by activating upstream kinases such as IKK, while NF-κB can also negatively regulate PTEN expression, thereby regulating the activity of the PI3K/Akt signaling pathway [50]. Therefore, the anti-inflammatory effect of BBR-CEH may not be achieved by targeting a single pathway, but by synergistically regulating these two pathways and their interaction networks. We will further explore this relationship and its impact on the mechanism of action of BBR-CEH in future research.

To elucidate the protective mechanism of BBR in *S. aureus*- and *E. coli*-induced mastitis, this study focused on the NF-κB signaling pathway. When bacteria invade mammary gland tissue and cause epithelial cell damage, damage-associated molecular patterns are released and recognized by corresponding receptors. This activates the downstream NF-κB signaling pathway and promotes the transcription of pro-inflammatory mediators. [51]. TJs are core structures that maintain the function of the BMB and are responsible for precisely regulating the exchange of substances between blood and milk [52]. The integrity of this structure is crucial for maintaining paracellular permeability [53]. Previous studies have shown that an increase in pro-inflammatory mediators can inhibit the transcription of TJ proteins [54]. In the bacteria-induced mastitis model, this study found that the levels of *TNF-α*, *IL-1β*, and *IL-6* were significantly elevated while the transcription of the TJ proteins *ZO-1*, *Claudin-4*, and *Occludin* was reduced. IF results showed disrupted ZO-1 localization, consistent with previous reports. Intervention with BBR-CEH effectively reversed the increase in these inflammatory factors and the decrease in TJ protein transcription. Concurrently, existing research has demonstrated that BBR can alleviate inflammatory responses by inhibiting the activation of the NF-κB pathway [55]. Further mechanistic study indicates that NF-κB pathway activation can inhibit TJ-associated protein transcription and disrupt cell polarity, thereby impairing TJ protein expression and localization [56]. This study validated this mechanism, showing that the p-p65/p65 ratio was significantly increased in the model group, indicating activation of the NF-κB pathway, along with abnormal transcription of TJ proteins. In contrast, BBR intervention significantly reduced the p-p65/p65 ratio and restored TJ protein transcription and ZO-1 distribution. These results suggest that bacteria-induced mastitis can activate the NF-κB pathway, promote inflammatory responses, and disrupt TJ structures. BBR may mitigate inflammatory damage by inhibiting this pathway, thereby restoring the function of the BMB.

To further elucidate the anti-apoptotic mechanism of BBR in mastitis induced by *S. aureus* and *E. coli*, this study focused on the PI3K/Akt signaling pathway. This pathway is a key regulator of apoptosis and plays an important role in the pathological progression and therapeutic intervention of mastitis [57]. Apoptosis, as a programmed cell death process, is primarily regulated by the p53 protein [58]. Activation of p53 upregulates the expression of the pro-apoptotic protein Bax while downregulating the anti-apoptotic protein Bcl-2, thereby increasing caspase-3 activity and triggering a cascade reaction that ultimately leads to apoptosis [59]. Previous studies have shown that inhibition of the PI3K/Akt signaling pathway reduces Akt phosphorylation levels, leading to insufficient activation of its downstream targets, such as murine double minute 2 (MDM2), a negative regulator of p53 [60]. Consequently, the stability and transcriptional activity of p53 increase, promoting its nuclear accumulation and expression, which ultimately induces apoptosis [61]. The results of this study demonstrated that in the bacterial invasion-induced mastitis model, the ratios of p-PI3K/PI3K and p-Akt/Akt were significantly decreased, indicating suppression of the PI3K/Akt pathway. Concurrently, p53 expression increased, and transcription of the pro-apoptotic protein *Bax* was elevated while transcription of the anti-apoptotic protein *Bcl-2* was reduced. TUNEL assays revealed obvious apoptotic phenomena consistent with the aforementioned studies. Although previous research has confirmed that BBR can regulate the PI3K/Akt pathway to exert protective effects in the hippocampus of gerbils [62], whether it functions through modulation of the PI3K/Akt pathway in a mouse model of mastitis remains unclear. This study found that BBR-CEH intervention significantly reversed the decrease in the p-PI3K/PI3K and p-Akt/Akt ratios, while also suppressing the elevation in p53 expression and apoptosis. These results suggest that BBR may alleviate bacterial-induced mammary epithelial cell apoptosis in mastitis by activating the PI3K/Akt signaling pathway and subsequently inhibiting the p53-dependent apoptotic pathway. This provides a new perspective on the role of BBR in the treatment of mastitis.

## 5. Conclusions

In summary, this study confirms that BBR-CEH exhibits efficient drug encapsulation and enhanced capabilities for transdermal delivery. Furthermore, it effectively alleviates LPS-induced mastitis by modulating the NF-κB and PI3K/Akt pathways. While the murine model provides supporting evidence for the properties and efficacy of BBR-CEH, anatomical, physiological, and pathogen differences between mice and cattle should be considered when translating these findings to bovine mastitis. Further validation using bovine mastitis models or ex vivo udder tissue would help support its potential for clinical use.

## Figures and Tables

**Table 1:** Design factor of orthogonal experiment.

Level	A (PC:HSPC)	B (%)	C (mg)
1	1:1	25	20
2	3:1	30	30
3	1:3	35	40

A: phospholipid ratio; B: ethanol concentration; C: cholesterol content.

**Table 2:** Composition of BBR-CEH formulations.

Ingredients	H_1_	H_2_	H_3_	H_4_	H_5_
BBR-free drug (mg)	-	-	-	-	7.5
BBR-loaded (mg)	7.5	7.5	7.5	7.5	-
Carbopol-934 (*w*/*w*)	0.5	1	1.5	-	1

**Table 3:** Physicochemical studies of BBR-CEH formulations (*n* = 3).

Parameter	H_1_ (0.5%)	H_2_ (1%)	H_3_ (1.5%)
Visual examination	homogenous	homogenous	homogenous
Viscosity (Pa·s)	39.80 ± 2.02	79.20 ± 1.50	More than 100
pH	5.81	5.94	6.02
Spreadability (mm)	66.7 ± 1.5	49.7 ± 1.2	36.3 ± 2.1
Drug content (%)	89.98 ± 0.99	92.50 ± 1.43	90.83 ± 0.41

## Data Availability

Data will be made available on request.

## Supplementary Materials

The following supporting information can be downloaded at: https://www.mdpi.com/article/10.3390/ani16091310/s1, TEXT S1: HPLC method; TEXT S2: Skin irritation Study; TEXT S3: Histopathological examination; TEXT S4: Western Blotting; TEXT S5: QRT-PCR; TEXT S6: VS; TEXT S7: EE; TEXT S8: Skin Irritation Study; Table S1: The table for skin irritation score; Table S2: Inflammatory scoring criteria of mammary gland tissues; Table S3: Primers Used for Quantitative Real-time PCR; Figure S1: Broken skin irritation reaction; Figure S2: Complete skin irritation response.

## Abbreviations

The following abbreviations are used in this manuscript:

*S. aureus*	*Staphylococcus aureus*
*E. coli*	*Escherichia coli*
TDDS	Transdermal drug delivery system
SC	Stratum corneum
PC	Phosphatidylcholine
HSPC	Phosphatidylcholine
BBR-CEH	BBR-loaded composite phospholipid ethosome hydrogel
BBR-CE	BBR-loaded composite phospholipid ethosome
EE	Entrapment efficiency
MBC	Minimum bactericidal concentration
BMB	Blood–milk barrier
HE	Hematoxylin eosin
IF	Immunofluorescence
PPI	Protein–protein interaction
TJ	Tight junction
