# Supplementary material for: Berberine-Loaded Composite Phospholipid Ethosome Hydrogels: A Therapeutic for Mastitis via Regulating the NF-κB and PI3K/Akt Signaling Pathway
Source: Animals (Basel). 2026 Apr 24;16(9):1310. doi: 10.3390/ani16091310 (PMC13163013; doi:10.3390/ani16091310)
Supplement: Supplementary file 1 [file animals-16-01310-s001.zip › animals-4208740-supplementary.pdf]

Supplementary Material

Berberine-Loaded Composite Phospholipid Ethosome Hydrogels: A Therapeutic for Mastitis via Regulating the NF-κB and PI3K/Akt Signaling Pathway

Binwen Zhang †, Zheng Wei †, Mei Yang, Xin Wang, Qiang Shan and Zheng Cao *

Heilongjiang Key Laboratory for Laboratory Animals and Comparative Medicine, College of Veterinary Medicine, Northeast Agricultural University, NO. 600, Changjiang Road, Harbin 150030, China; 13569501808@163.com (B.Z.); m15003567979@163.com (Z.W.); 17513204419@163.com (M.Y.); 17642186580@163.com (X.W.)

* Correspondence: neaucz@neau.edu.cn

† These authors contributed equally to this work.

TEXT S1: HPLC method

The HPLC system used for the analysis was an Agilent 1260 series (Agilent Technologies, Santa Clara, CA, USA). The mobile phase consisted of acetonitrile (A) and 0.1% phosphoric acid (B) with gradient elution as follows: 0–1 min, 10% A; 1–6 min, 10–90% A; 6–8 min, 90% A; 8–8.2 min, 90–10% A; 8.2–10 min, 10% A. The use of reversed-phase C18 columns (5 μm, 4.6 x 100 nm). The detection wavelength was 344 nm, the flow rate was 0.7 mL/min, and the injection volume was 10 μL. To guarantee the reliability of the HPLC method for the analysis of BBR, the precision, reproducibility and stability of the HPLC method were verified.

TEXT S2: Skin irritation Study

Tab. S1 The table for skin irritation score

Erythema Formation	Score	Edema Formation	Score
No erythema	0	No edema	0
Slight erythema	1	Slight edema	1
Moderate erythema	2	Moderate edema	2
Severe erythema	3	Severe edema	3
Severe erythema to slight eschar formation	4	Severe edema (raised more than 1 mm and extending beyond area of exposure)	4

TEXT S3: Histopathological examination

Three skilled pathological section analysts were invited to score the histopathological changes including the degree of neutrophil aggregation, acinar integrity, and changes in acinar wall thickness by selecting five fields of view per section. The detailed scoring criteria are provided in the Tab. S2.

Tab. S2 Inflammatory scoring criteria of mammary gland tissues

Feature	Description	Score
Neutrophil aggregation	Normal injury	0
	Mild injury	1
	Moderate injury	2
	Severe injury	3
	Extreme injury	4
Acinar integrity	Normal injury	0
	Mild injury	1
	Moderate injury	2
	Severe injury	3
	Extreme injury	4
Acinar wall thickness	Normal injury	0
	Mild injury	1
	Moderate injury	2

Severe injury	3
Extreme injury	4

TEXT S4: Western Blotting

First, proteins were isolated from mouse mammary gland tissue using a protein extraction kit (Beyotime, Shanghai, China). Protein density was determined using the BCA Protein Assay Kit (Beyotim, Shanghai, China). Proteins (30-50 μg) were detached by 8-15% sodium dodecyl sulfate polyacrylamide gel electrophoresis and shifted to PVDF membranes (0.45 μM, Millipore, Massachusetts, USA). These membranes were interdicted with QuickBlock TM Western Sealant (Beyotime, ShangHai, China) at 25◦ C for 15 min, and stimulated overnight at 4◦ C with primary antibody: p-PI3K (1:1000, WanLei, Shenyang, China), PI3K (1:1000, WanLei, Shenyang, China), Akt (1:500, WanLei, Shenyang, China), p-Akt (1:500, WanLei, Shenyang, China), p65 (1:500, WanLei, Shenyang, China), p-p65 (1:500, WanLei, Shenyang, China), p53 (1:1000, WanLei, Shenyang, China) and β-actin(1:3000, Abclonal, Massachusetts, USA). The primary antibodies were subsequently localized with goat anti-rabbit IgG (1:5000, Affinity, Jiangsu, China) for 2h at 37◦C. Subsequently incubated with goat anti-rabbit IgG (1:5000, Affinity, Jiangsu, China) for 2 h at 37◦C. by the BeyoECL Star kit (Beyotime, Shanghai, China). Finally, the protein bands were analyzed for relative quantification using Amersham Imager 600 (Fairfield, Connecticut, USA).

TEXT S5: Quantitative Real-Time Polymerase Chain Reaction (QRT-PCR)

We extracted total RNA from mammary tissue using TRIzol reagent (Invitrogen, California, USA). The extracted RNA was transcribed into cDNA following the steps of the reverse transcription kit (VazymE, Nanjing, China). We detected gene expression using the ABI 7500 system, and finally mRNA expression of *TNF-α, IL-1β, IL-6 Bax, Bcl-2, ZO-1, Occludin* and *Claudin-4* were relatively quantified using β-actin (Shangon Bio, Shanghai, China) as a normalizer.

Table S3. Primers Used for Quantitative Real-time PCR

Gene	sequences(5'to3')
IL-1β— f	GTGGCTGTGGAGAAGCTGTGG
IL-1β — r	CGGAGCCTGTAGTGCAGTTGTC
IL-6— f	ACTTCCATCCAGTTGCCTTCTTGG
IL-6 — r	TTAAGCCTCCGACTTGTGAAGTGG
TNF-α - f	GATGGGTTGTACCTTGTCTACT
TNF-α —r	CTTTCTCCTGGTATGAGATAGC
Bax- f	CTT TTGCTTCAGGGTTTCA
Bax-r	GCTCAGCTTCTTGGTGGAT
Bcl-2 -f	ATGACTTCTCTCGGCGCT
Bcl-2-r	CGGTTCAGGTACTCGGTCAT
ZO-1-f	GAGTGGACTATCAAGTGAGCCTAA
ZO-1-r	ATCCAAGTTGCTCGTCAATCTAA
Occludin-f	CTATGGGACAGGGCTCTTTGGA
Occludin-r	AGGAAGCGATGAGAGAGAGAGAGAC
Claudin-4-f	GTGCCTTGCTCACCGAAAC
Claudin-r	CCACCACTGCCCAAACCT
β-actin— f	GTTGGAGCAAACATCCCCCA
β-actin— r	ACGCGACCATCCTCCTCTTA

TEXT S6: Orthogonal test results of formulation optimization (VS)

FN	A (PC：HSPC)	B (%)	C (mg)	VS (nm)
1	1:1	25	20	181.0
2	1:1	30	30	169.4
3	1:1	35	40	172.9
4	3:1	30	40	186.3
5	3:1	35	20	180.1
6	3:1	25	30	176.2
7	1:3	35	30	433.6
8	1:3	25	40	447.4
9	1:3	30	20	426.4
K_1	523.3	804.6	787.5	
K_2	542.6	782.1	779.2	
K_3	1654.8	786.6	806.6	
R	1131.5	22.5	27.4	

FN: formulation number; A: phospholipid ratio; B: ethanol concentration; C: cholesterol content; VS: vesicle size

TEXT S7: Orthogonal test results of formulation optimization (EE)

FN	A (PC：HSPC)	B (%)	C (mg)	EE (%)
1	1:1	25	20	91.48
2	1:1	30	30	95.76
3	1:1	35	40	94.03
4	3:1	30	40	91.72
5	3:1	35	20	93.53
6	3:1	25	30	95.06
7	1:3	35	30	97.35
8	1:3	25	40	97.97
9	1:3	30	20	97.42
K_1	2.8127	2.8451	2.8243	
K_2	2.8031	2.8491	2.8817	
K_3	2.9274	2.8490	2.8372	
R	0.1243	0.004	0.0574	

FN: formulation number; A: phospholipid ratio; B: ethanol concentration; C: cholesterol content; EE: entrapment efficiency

TEXT S8：Skin Irritation Study

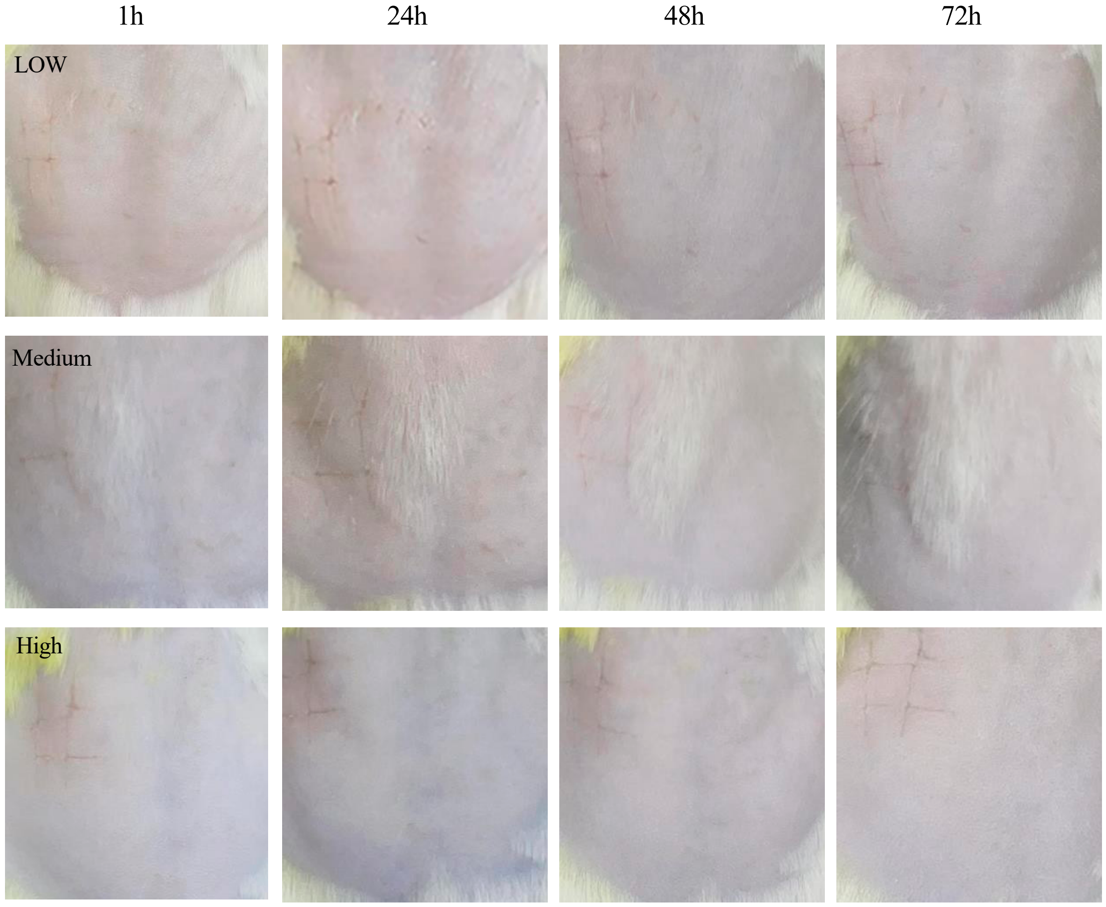

Fig. S1 Broken skin irritation reaction

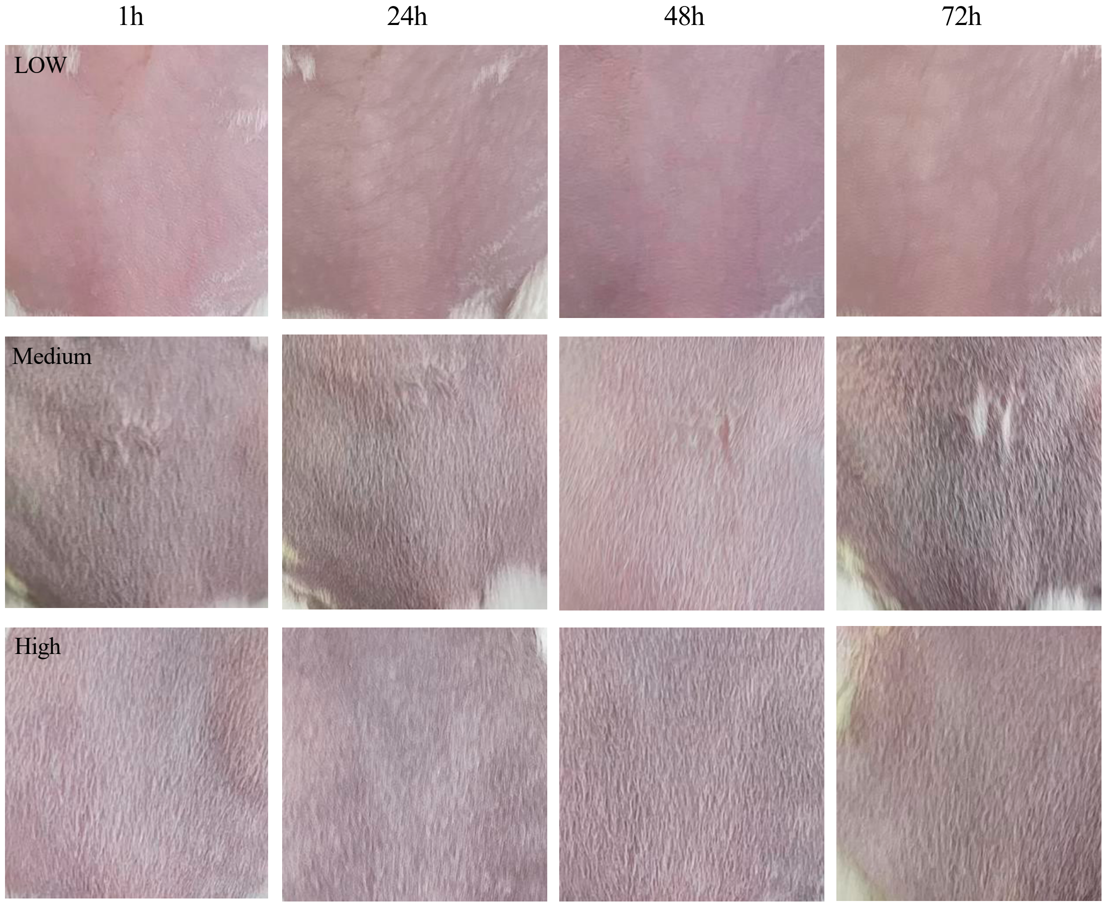

Fig. S2

Complete skin irritation response
